# Graphene-based nanocomposites as gamma- and X-ray radiation shield

**DOI:** 10.1038/s41598-024-69628-5

**Published:** 2024-08-16

**Authors:** Karolina Filak-Mędoń, Krzysztof W. Fornalski, Michał Bonczyk, Alicja Jakubowska, Kamila Kempny, Katarzyna Wołoszczuk, Krzysztof Filipczak, Klaudia Żerańska, Mariusz Zdrojek

**Affiliations:** 1 Faculty of Physics, Warsaw University of Technology, Koszykowa 75, 00-662 Warszawa, Poland; 2 Silesian Centre for Environmental Radioactivity, Central Mining Institute – National Research Institute (GIG-PIB), Plac Gwarków 1, 40-166 Katowice, Poland; 3 Central Laboratory for Radiological Protection (CLOR), Konwaliowa 7, 03-194 Warszawa, Poland; 4 Department of Quality Control and Radiation Protection, Medical University of Łódź, 92-216 Łódź, Poland

**Keywords:** Ionizing radiation, Graphene, X-ray, Gamma, Radiation shield, Attenuation coefficient, Nanocomposites, Nanoscience and technology, Graphene, Materials science

## Abstract

Commonly used materials for protection against ionizing radiation (gamma and X-ray energy range) primarily rely on high-density materials, like lead, steel, or tungsten. However, these materials are heavy and often impractical for various applications, especially where weight is a key parameter, like in avionics or space technology. Here, we study the shielding properties of an alternative light material—a graphene-based composite with a relatively low density ~ 1 g/cm^3^. We demonstrate that the linear attenuation coefficient is energy of radiation dependent, and it is validated by the XCOM model, showing relatively good agreement. We also show that the mass attenuation coefficient for selected radiation energies is at least comparable with other known materials, exceeding the value of 0.2 cm^2^/g for higher energies. This study proves the usefulness of a commonly used model for predicting the attenuation of gamma and X-ray radiation for new materials. It shows a new potential candidate for shielding application.

## Introduction

The development of new materials serving as a shield for ionizing electromagnetic (EM) radiation is crucial for various industrial and scientific areas, like radiation medicine, the nuclear industry, or the aerospace sector^1–4^ . High energy photons, namely gamma and X-ray, interact with matter in a few different ways: as Thomson scattering, Rayleigh scattering and photoelectric effect for lower energies, and as Compton scattering for medium energies, and pair creation for high energies. All those effects are described by their proper cross-section values (a measure of the probability that a scattering process will occur), which are higher for heavier elements^5–8^. That is why materials like lead, concrete or steel are commonly used as radiation shields against ionized radiation^9–11^. Although these popular heavy materials are commonly used for ordinary shielding tasks, they are not practical in many applications because of their heterogeneity, moisture variation^12^ and lack of plasticity, overshadowing their benefits^13,14^. On the other hand, light element-based materials, like carbon, have much smaller values of cross sections because their nucleus contains much fewer protons with much fewer electrons around them, making them much less useful as a shield against ionized radiation. Therefore, recent research focuses on materials that can be used as efficient radiation shields while exhibiting good mechanical durability and flexibility as well as low weight^4,15,16^.

Most lightweight materials have poor attenuation properties in the gamma and X-ray energy ranges. However, novel nanocomposites containing e.g. bismuth oxide nanoparticles^17^, cobalt-doped titania^18^, carbon nanotubes or graphene oxide flakes^19^ or their combination, can present a new approach for high energy radiation shields, showing moderate effectiveness. A recent review article discussed the topic of carbon-based (graphite, graphene oxide) materials for radiation shielding in X-ray and gamma ranges, highlighting novel ideas for radiation protection applications in medicine and industry^19^. In one of our previous works, we studied the interaction of bare thin graphene film with ionizing radiation, demonstrating a potential candidate for serving as a light and flexible EM shield^20^. But composite based on pure graphene flakes has not yet been reported to show shielding properties in the ionizing energy ranges.

In this work, we explore the shielding properties of graphene/acrylonitrile butadiene styrene (ABS) composites in several distinct radiation energies in the X-ray and gamma regimes. We show that the shielding efficiency (mass/linear attenuation coefficient) depends on the radiation energy and, in some cases, outperforms other known materials. Finally, we have validated the experimental results with a commonly used XCOM theoretical model^21–24^.

## Materials and methods

### Composite fabrication

Graphene nanoplatelets (GNPs) were obtained from Sigma-Aldrich. GNPs were supplied in powder form, with an average lateral dimension of 25 µm and a surface area ranging from 50 to 80 m^2^/g. Individual flakes have a thickness in the range of 1–5 nm (verified by AFM—not shown here). Commercially available ABS was utilized as the polymer matrix.

The fabrication process of the nanocomposite involved two simple steps. Firstly, the GNPs were mixed with the ABS matrix using a classical mechanical mixer. The weights of the polymer matrix and the GNPs were calculated to achieve the 10 weight percentage of the graphene filler loading. The mixing process was carried out for 60 min at a rotational speed of 100 rpm, using a three-dimensional mixer to ensure a homogeneous filler distribution within the matrix. Subsequently, the polymer/GNP mixture was subjected to hot pressing using a laboratory platen press (LabEcon 300 Fontijne). The purpose of hot pressing was to consolidate the powder material into solid samples (see Fig. 2a). The hot-pressing parameters included a heating temperature of 190 °C and a downforce of 250 kN. The thickness of the samples fabricated via the hot-press method was controlled by employing a spacer during the process. Samples with different thicknesses (~ 3 and 15 mm) and a diameter of 60 mm have been used for the radiation measurements.

The composition of nanocomposite indicates the proportion of each component in the material, with graphene nanoplatelets making up 10% of the total weight, styrene comprising 45%, acrylonitrile accounting for 27%, and butadiene contributing 18% to the overall weight. The density of the nanocomposite determined using hydrostatic weighing at room temperature of 21.7 °C was 1.064 g/cm^3^. This information is used later in the XCOM calculation.

### Radiation measurements

**Figure 1:**
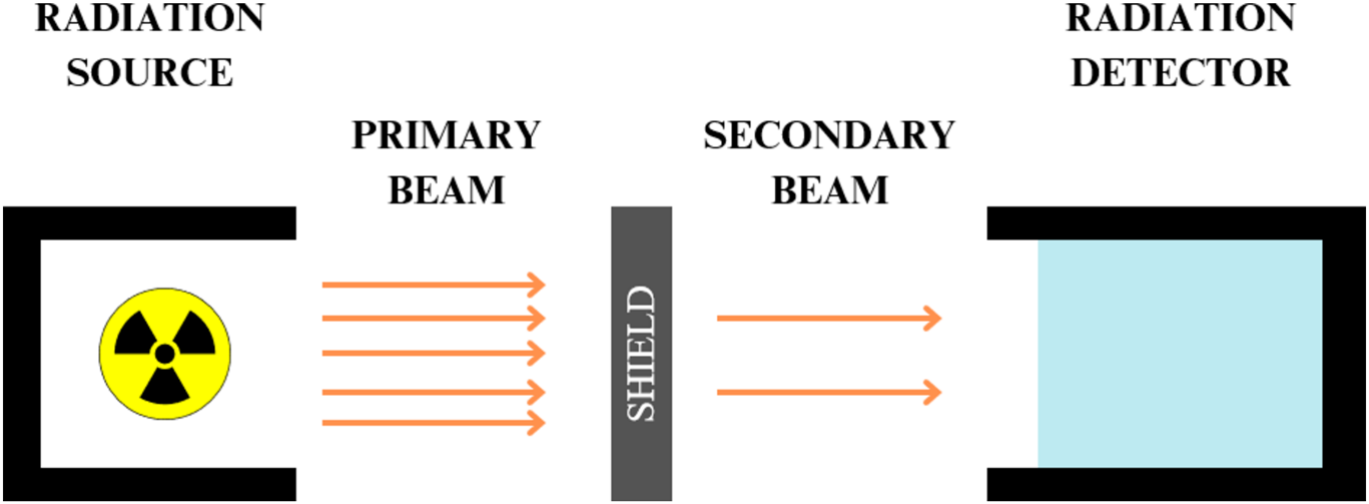
Experimental Setup for Gamma Radiation Measurements.

Measurements of gamma radiation attenuation were carried out in three independent laboratories: Silesian Centre for Environmental Radioactivity (Central Mining Institute – National Research Institute, GIG, Katowice, Poland), Department of Individual Monitoring and Calibration (Central Laboratory of Radiological Protection, CLOR, Warsaw, Poland), and Department of Quality Control and Radiation Protection (Medical University, UM, Łódź, Poland). Figure 1 depicts the general schematic diagram of the radiation measurement setup.

In the GIG laboratory, the experimental setup consisted of a spectrometric system, a radioactive point source (Pb-210, Ba-133 and Cs-137) in a lead collimator, and a shielding house (15 cm of aged lead covered by tin and copper). The collimator thickness was 5 cm. The diameter of the hole in the collimator was 1 mm. l Detector crystal parameters: active diameter 81 mm, active area 5000 mm^2^, thickness 31 mm, window material—carbon epoxy, window thickness—0.5 mm. The graphene samples were placed directly on the detector. The measurements were conducted by considering the number of counts in the appropriate photopeak for analysis and measurement times ranged from 5000 to 25,000 s, depending on the peak shape and count statistics. The goal was to obtain a peak with an uncertainty statistic of less than 5%. During the measurement, the sources had the following activities: Pb-210 approximately 138 kBq, Cs-137 approximately 130 kBq, and Ba-133 approximately 16 kBq.

In CLOR, the measurements were conducted using the TM23361 ionization chamber. The samples with different thicknesses were positioned between the source and the ionization chamber, ensuring the entire chamber was within the attenuated beam. The measurements were conducted in dose measurement mode. Seven one-minute exposures were performed without a sample to determine the reference value. It was made at the beginning and end of measurements to be sure nothing had changed during this time. The mean value (14 points) was taken as a reference value. Between these measurements, the procedure was repeated using samples. Additionally, the CLOR laboratory conducted the graphene nanocomposite examination using an X-ray generator model HF 320 produced by PANTAK with the X-ray tube MXR-350/26. The Cs-137 activity source is 410 ± 40 GBq.

The research conducted by the laboratory at the Department of Quality Control and Radiation Protection at the Medical University in Łódź focused on the use of three isotopes as sources of radiation: cobalt, barium, and cesium with photon energies of 122, 356, and 662 keV, respectively. The sources had the following activities: Co-57 approximately 175.5 MBq, Cs-137 approximately 370 kBq, and Ba-133 approximately 9912 kBq. For each radiation source, a calibration measurement was performed to verify the correct positioning of the detector’s energy window. Due to the limited energy resolution of the scintillation probe, photons within a specific energy range were recorded for each of the: Co-57: 104–156 keV; Ba-133: 320–392 keV; Cs-137: 562–761 keV. During the measurements, additional samples were added: for each material and thickness (~ 3 and 15 mm), three 1-min beam intensity measurements were taken, and the average count rate was used to calculate the gamma radiation attenuation coefficient.

In all three laboratories, the radiation measurements on the ABS/GNP composites were conducted in gamma and X-ray ranges. In each case the ionizing radiation detector compared photon counts with (*N(x)*) and without (*N*_*0*_) ABS/GNP composite layer, to determine linear (*µ*_*x*_) or mass (*µ*_*d*_) attenuation coefficient^25^:

1 $$ \frac{N\left( x \right)}{{N_{0} }} = e^{{ - \mu_{x} x}} = e^{{ - \mu_{d} \rho x}} $$

where *x* is the absorber’s thickness, and *ρ* its density. This was necessary to verify its theoretical calculations by the popular XCOM model^21^ which can determine crucial radiation properties of the vast majority of elements (or their mixture). The XCOM model is a widely used method for the prediction of high-energy photons interaction with matter^21^.

Radiation protection applications are usually based on the mentioned linear or mass attenuation coefficient. However, in some medical cases, a more practical factor is HVL (half-value layer), which means the material that gives a 50% reduction of the radiation beam and is calculated by the following equation^26^:

2 $$ HVL = \frac{\ln \left( 2 \right)}{{\mu_{x} }} $$

where ${\upmu }_{\text{x}}$ (cm^−1^) is the linear attenuation coefficient of the absorber, mentioned earlier.

## Results and discussion

**Figure 2:**
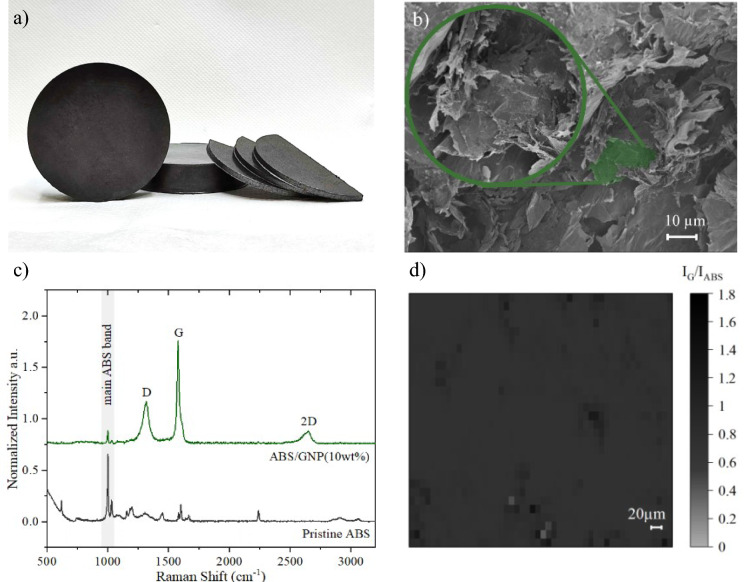
(**a**) Hot-pressed nanocomposite samples containing 10 wt% with a different dimension, (**b**) typical SEM image of graphene flakes immersed in the composite matrix, (**c**) normalized Raman spectra of pristine ABS and ABS/GNP nanocomposite showing the peaks corresponding to graphene and ABS, d) Raman map reflecting the relative intensity ratio of peaks corresponding to graphene and polymer base from the measured ABS/GNP (10wt%) composite sample.

Scanning electron microscope (Raith e-line +) provides an indication of the lateral dimension of the GNPs (see Fig. 2b) immersed in a composite as filler. For the analysis of the chemical composition and structure of the composite material, Raman Spectroscopy was employed (Renishaw inVia, 785 nm line, 50 × long-distance objective). In Fig. 2c, the Raman spectrum for pure ABS (bottom spectrum) and the ABS/GNP nanocomposite (top spectrum) are presented. For ABS, the main peak visible at 1001 cm^−1^ can be attributed to the breathing vibration of the benzene ring in the styrene-acrylonitrile part of ABS^27,28^. The typical collected Raman spectrum of the ABS/GNPs nanocomposite, apart from the main polymer peak (with intensity lower than in the case of pure polymer), shows typical graphene bands (D, G and 2D), at 1315 cm^−1^, 1578 cm^−1^ and 2645 cm^−1^, respectively^29–31^. Statistical Raman mapping shows that the graphene-related bands are detected in every place of the sample (based on the relative intensity of graphene G peak), thus confirming the uniform dispersion of graphene flakes within the polymer matrix (Fig. 2d). Further evidence of the graphene filler homogenization is reflected in the volume resistivity measurements reaching the value of ~ 180 S/m (measured using a single-post dielectric resonator QWED). Finally, we note that the EMI shielding properties for our graphene-composite have already been confirmed for a very broad range of frequencies, from megahertz up to terahertz radiation^32^.

**Figure 3:**
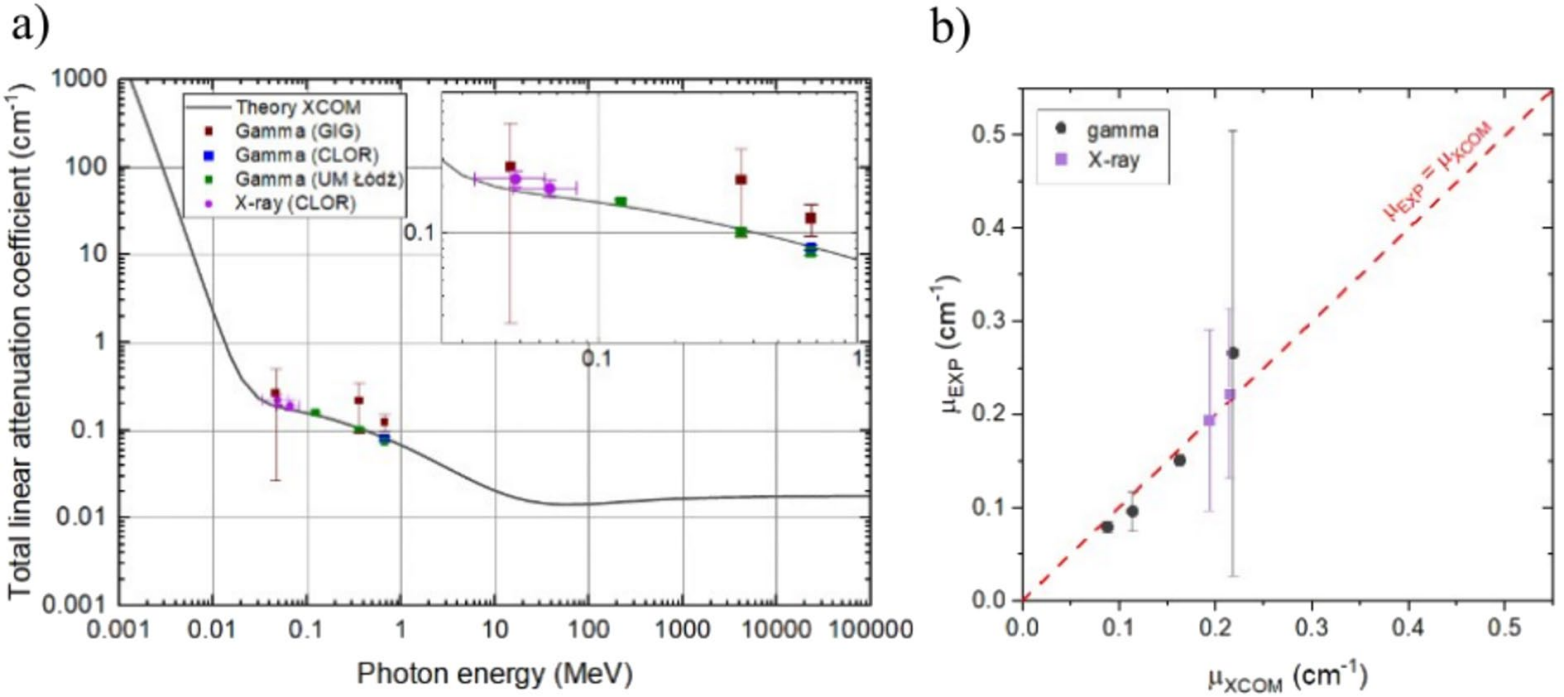
(**a**) Comparison of the beam (linear) attenuation coefficient for the Lead (Pb-210), Cobalt (Co-57), Barium (Ba-133), and Cesium (Cs-137) isotopes obtained by four different laboratories. Additionally, round points cover the results of X-ray irradiation for two different energies. The black solid line represents theoretical calculations (XCOM model). All uncertainties represent two standard deviations, (**b**) experimental and expected (XCOM) half value layer (cm) as a function of photon energy with arbitrary linear fit.

For the experimental investigations of gamma radiation of the ABS/GNP nanocomposites, we utilized four gamma-ray isotopes: Lead (Pb-210), Cobalt (Co-57), Barium (Ba-133), and Cesium (Cs-137) with a gamma photon energy of 46, 122, 356 and 662 keV, respectively. Every laboratory used their own radioactive sources. All results—linear attenuation coefficient—are presented in Table 1 and Fig. 3 (squares), together with the theoretical predictions based on the XCOM model^21^.

**Table 1:** Comparison of linear (µ_x_) attenuation coefficients for four different isotopes, and their theoretical predictions based on the XCOM model.

Radiation source	Linear attenuation coefficient µ_x_ (cm^−1^)			
	GIG	CLOR	UM Łódź	XCOM (theory)
Pb-210 (46 keV)	0.266 ± 0.239	–	–	0.218
Co-57 (122 keV)	–	–	0.151 ± 0.006	0.163
Ba-133 (356 keV)	0.220 ± 0.126	–	0.096 ± 0.005	0.114
Cs-137 (662 keV)	0.125 ± 0.028	0.0866 ± 0.003	0.071 ± 0.003	0.088

All uncertainties represent two standard deviations.

We note that the methodology employed by GIG was associated with higher measurement uncertainty than the methodologies used by the laboratories (CLOR and UM). Specifically, GIG utilized a germanium detector known for its relatively lower sensitivity relative to the ionization chamber used by the other laboratories. Furthermore, the radioactive sources Pb-210 and Ba-133 applied by GIG exhibited lower activity levels than anticipated for optimal measurement conditions. Despite these differences in methodology and source activity, the results obtained by GIG exhibited minor deviations from those obtained by CLOR and UM. Importantly, these deviations were within the anticipated uncertainty range of two standard deviations established for this comparative study, except the point for highest energies (Fig. 3a), which follows theoretical prediction with three standard deviations.

Additionally, graphene nanocomposites were irradiated by two different X-ray sources in the CLOR laboratory: N-60 (with an average energy of 48 keV) and N-80 (with an average energy of 65 keV). The spectra were generated based on the standard ISO 4037-1:2019^33^. The attenuation coefficients are presented in Table 2 and in Fig. 3a (purple dots).

**Table 2:** Linear attenuation coefficients (μ_x_) obtained by X-ray irradiation for two different X-ray machines in CLOR laboratory.

X-ray machine	Average photon energy (keV)	Linear attenuation coefficient μ_x_ (cm^−1^)	
		Experiment	Theory (XCOM)
N-60	47.9 ± 12.15	0.223 ± 0.027	0.215
N-80	65.2 ± 14.95	0.194 ± 0.026	0.193

Theoretical calculations were performed by the method described in Supplementary Information.

All attenuation coefficients are in the range of 0.07–0.5 cm^−1^ and are consistent with the theoretical prediction (XCOM model), however, some data scattering noise is observed, especially in the case of GIG results. The best agreement between theory and experiment is observed for gamma sources from UM and CLOR laboratories. The X-ray data points correspond to the mean energy values of the standard X-ray spectra, with horizontal error bars representing two standard deviations calculated from the energy distributions described in Supplementary information. Overall, these results show that the XCOM model is a useful tool for precise calculations of radiation attenuation coefficients and cross-sections of carbon-based composites.

All points are located in the energy region where Compton scattering dominates (with a weak influence of the photoelectric effect for the lowest energies). Electron–positron pair production is forbidden for this energy range. This means that all analyzed samples interacted with gamma and X-ray radiation via electrons interaction only, which may be important from the point of view of the specified electron structure in graphene. The XCOM model assumes that each absorbing material is a composition of single chemical elements with a standard electron structure. This means that different electron structures, especially with different bounding energies, can slightly influence the results where Compton scattering and photoeffect dominate (both are photon-electron interactions). Due to the graphene structure and the possibility of its modification (e.g., by negative charging of graphene), there is a chance to change its attenuation coefficient^20^. This does not mean that an analogical situation was observed here—but the trend observed in Fig. 3a leaves room for further discussion.

The same results can be presented in a simple comparison between experimental and expected results of the linear attenuation coefficient for gamma and X-ray radiation (see: Fig. 3b). The experimental data represent the averaged values of the linear coefficient obtained from various laboratories. The results indicate that the experimental data is well-aligned with the theoretical data.

**Figure 4:**
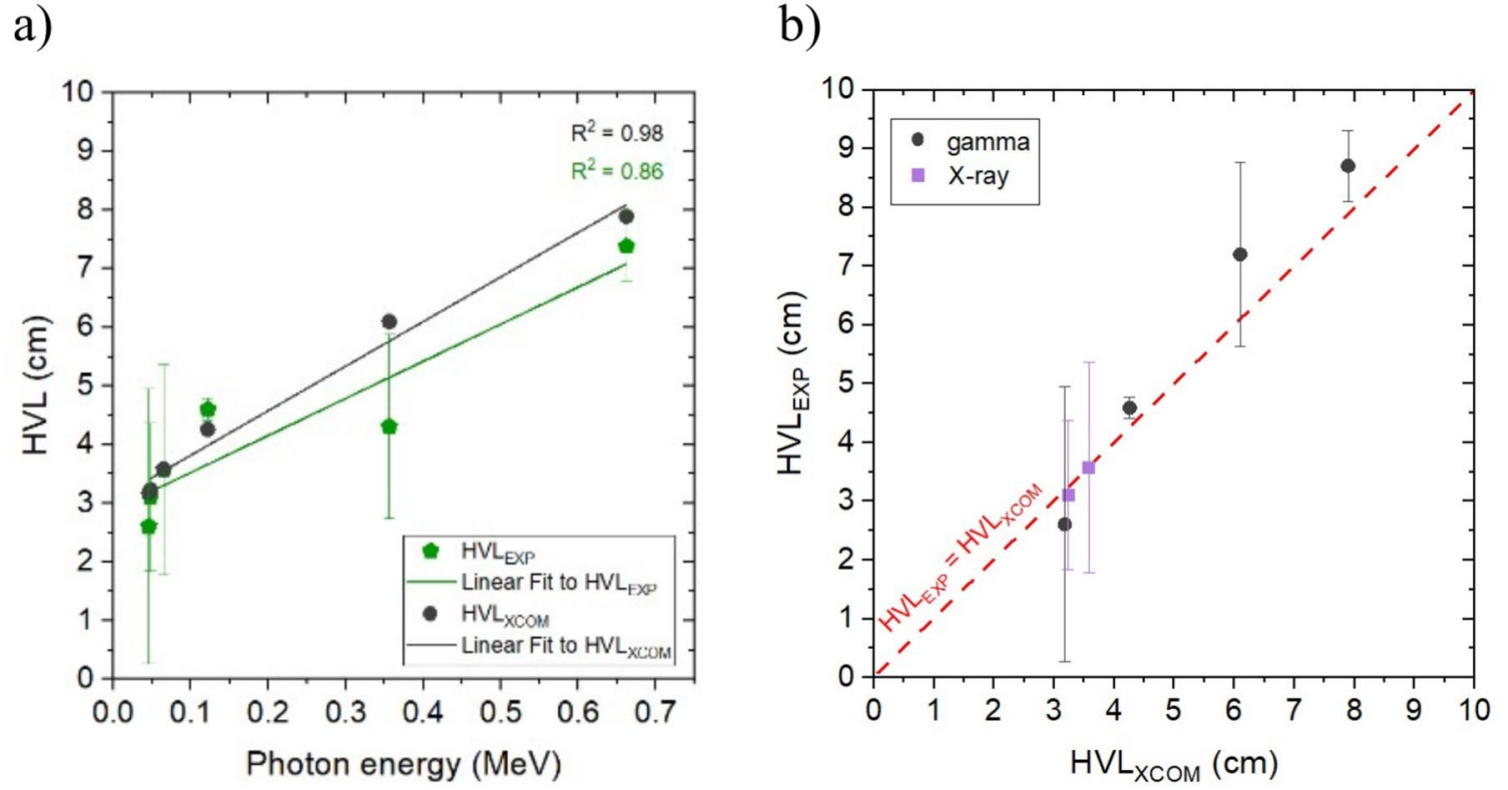
(**a**) Comparison of experimental linear attenuation coefficient (cm^−1^) for gamma and X-ray radiation with theoretical data from XCOM, (**b**) experimental and theoretical HVL (cm) comparison.

Typically, the predominant approach used to assess the overall filtration of material to the quality of the radiation beam, often referred to as penetrating energy, is defined by the half-value layer (Eq. 2). Therefore, we show HVL values, both for experimental and theoretical results in Fig. 4a. A noticeable correlation between photon energy and HVL suggests that higher energies penetrate more and require a greater thickness of an absorber to reduce radiation intensity by half. Also, the comparison of HVL values allows for additional validation of the theoretical model, confirming its consistency (see Fig. 4b).

**Figure 5:**
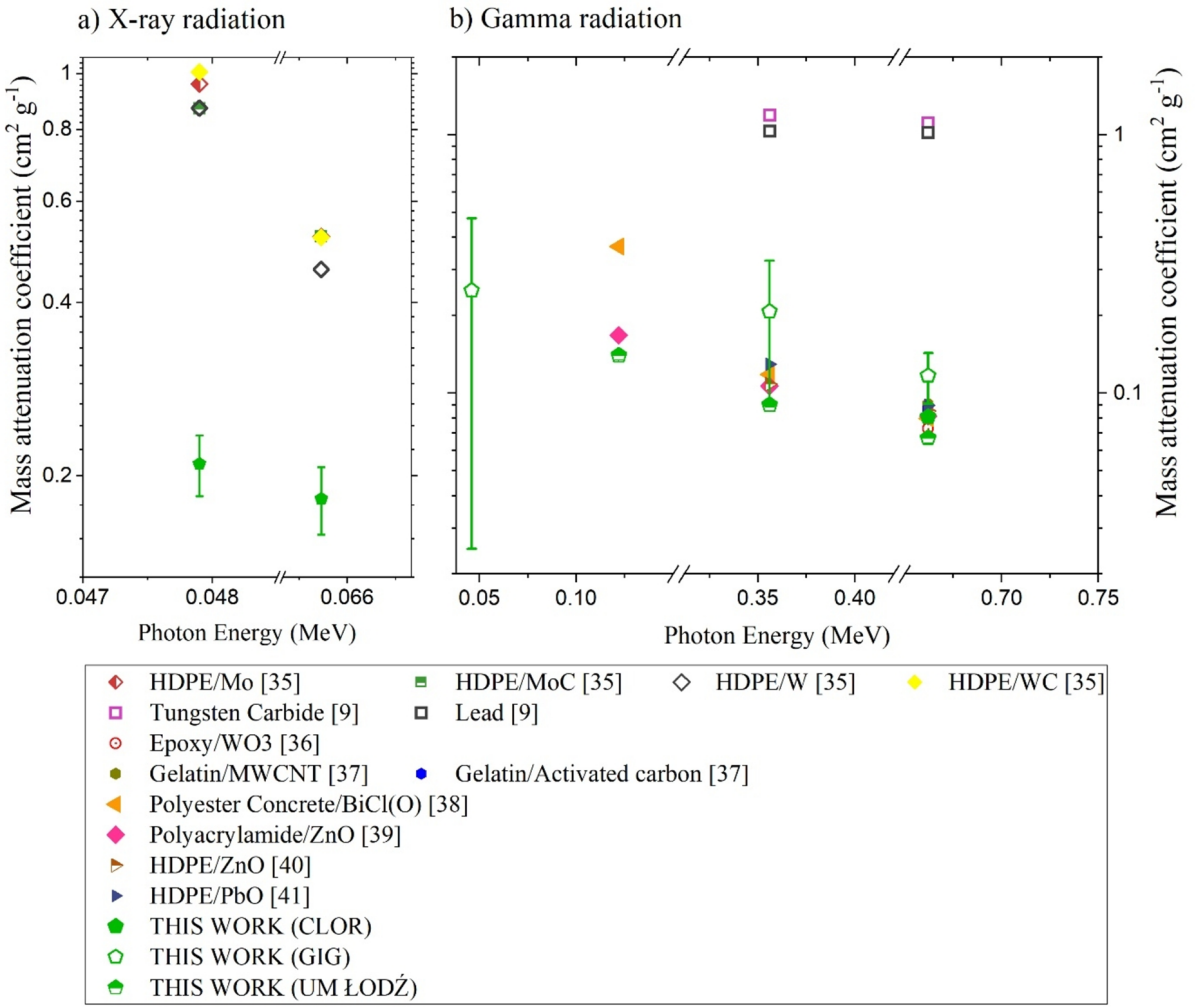
Comparison of the mass attenuation coefficient for (**a**) X-ray and (**b**) gamma radiation for nanocomposite samples from the presented study and samples developed by other researchers (see: Supplementary information, Table S3).

An important criterion for validation of the shielding performance of our composite compared with other existing materials is the mass attenuation coefficient (linear attenuation coefficient divided by the material density). For the analysis, an averaged attenuation coefficient value from various laboratories for a specific photon energy was employed. The mass attenuation coefficients for two radiation ranges for selected materials and composites^9,34–40^ are presented in Fig. 5. For energies higher than 100 keV (that are widely used in industrial radiation sources), the radiation properties of our samples are better than other nanocomposite materials (GIG) or at least comparable (CLOR, UM).

Another validation of the shielding performance of our composite is the comparison with classical materials used as typical shields in radiation protection, namely aluminum, iron, copper, and lead. We used the data delivered by the Department of Quality Control and Radiation Protection (UM) for three photon energies mentioned earlier: 122, 356, and 662 keV. The result of that experimental investigation is presented in Supplementary information in Table S3.

## Conclusions

We demonstrated pure graphene-based nanocomposites that can serve as potential radiation shields against ionizing radiation in the gamma and X-ray ranges, boasting a mass attenuation coefficient exceeding 0.2 cm^2^/g. Experimental investigations show that their radiation properties are consistent with the theory (XCOM). This could enable the construction of e.g., appropriate radiation shields for industry and medicine, as the attenuation coefficients (linear or mass) are necessary to calculate the absorbing material thickness.

### Supplementary Information

Supplementary Information.

## Data Availability

The datasets generated during and/or analyzed during the current study are available from the corresponding author on reasonable request.
