# Supplementary material for: Graphene-based nanocomposites as gamma- and X-ray radiation shield
Source: Sci Rep. 2024 Aug 16;14:18998. doi: 10.1038/s41598-024-69628-5 (PMC11329645; doi:10.1038/s41598-024-69628-5)
Supplement: Supplementary file 1 — Supplementary Information. [file 41598_2024_69628_MOESM1_ESM.docx]

**Supplementary information**

**Graphene-based nanocomposites as gamma- and X-ray radiation shield**

Karolina Filak-Mędoń^1,^*^,†^ Krzysztof W. Fornalski^1, †^, Michał Bonczyk^2^, Alicja Jakubowska^3^, Kamila Kempny^3^, Katarzyna Wołoszczuk^3^, Krzysztof Filipczak^4^, Klaudia Żerańska^1^, Mariusz Zdrojek^1^

1 Faculty of Physics, Warsaw University of Technology, Koszykowa 75, 00-662 Warszawa, Poland

2 Silesian Centre for Environmental Radioactivity, Central Mining Institute (GIG), Plac Gwarków 1, 40-166 Katowice, Poland

3 Central Laboratory for Radiological Protection (CLOR), Konwaliowa 7, 03-194 Warszawa, Poland

4 Department of Quality Control and Radiation Protection, Medical University of Łódź, 92-216 Łódź, Poland

* corresponding author: [karolina.filak.dokt@pw.edu.pl](mailto:karolina.filak.dokt@pw.edu.pl)

^†^ authors contributed equally

**Methodology for calculation of the effective mass attenuation coefficient for X-ray spectra**

The XCOM model ^21^ provides theoretical values of mass attenuation coefficients for different chemical elements and their mixtures. Exact values of energy are appropriate for isotopic radiation sources. However, these values are not directly applicable to radiation produced by X-ray generators, which emit a broad continuous spectrum. This appendix presents a straightforward approach to approximate effective mass attenuation coefficients for two radiation qualities defined by the ISO 4037-1:2019 international standard ^33^. The method can be readily adapted for other radiation qualities and materials as required.

The effective mass attenuation coefficients were calculated as the arithmetic mean of mass attenuation coefficients, weighted by radiation intensity at the corresponding energy values. The intensity spectra were calculated using the TASMIP algorithm ^41^, which provides photon energy spectra for a tungsten-anode X-ray generator at a given tube potential. The TASMIP algorithm played a crucial role in precisely predicting the quantum fluence across various X-ray beams. Leveraging the TASMIP interpolating polynomial tungsten anode spectral model, X-ray spectra were computed meticulously, with calculations made in increments of 1 keV, spanning from 10 keV to 80 keV. The outcome of the TASMIP algorithm yielded X-ray spectra that not only matched but also encompassed both qualitative and quantitative attributes of the X-ray system. All theoretical mass attenuation coefficients for specific energy values were computed using the XCOM model ^21^.

The measurements described in the paper were carried out using two standard radiation qualities labelled N-60 and N-80. The characteristics of the spectra, as well as the production method, are described in the ISO 4037-1:2019 standard ^33^. In principle, the radiation quality is achieved through a suitable selection of X-ray tube potential and filtration.

Table S1. Characteristics of the used standard radiation qualities for two average X-ray energies

| Tube potential (kV) | Short name | Mean Energy (keV) | Resolution % | Recommended inherent filtration  *x_Al_* | Additional filtration thickness  *x_Cu_* |
| --- | --- | --- | --- | --- | --- |
| 60 | N-60 | 47.9 | 36 | 4 mm Al | 0.6 mm Cu |
| 80 | N-80 | 65.2 | 32 | 4 mm Al | 2 mm Cu |

Firstly, the TASMIP spectra calculator was employed to compute photon energy spectra for two tube potential values as per Supplementary Table S1, without filtration. Subsequently, the impact of the corresponding filtration was determined using the exponential attenuation law. The values of linear attenuation coefficients for aluminum, µ_Al_(E), and copper, µ_Cu_(E), were sourced from the XCOM model. Finally, the photon energy spectrum is expressed by the following formula:

| $I\left( E,V \right)=I_{TASMIP}\left( E, V \right)\cdot\exp\left( -\mu_{Al}\left( E \right)x_{Al}\left( V \right) \right)\cdot\exp\left( - \mu_{Cu}\left( E \right)x_{Cu}\left( V \right) \right)$ | (S1) |
| --- | --- |

where I(E,V) – final radiation intensity at specific energy for a particular radiation quality, I_TASMIP_(E) – unfiltered radiation intensity at a specific energy and tube potential, computed with the TASMIP spectra calculator, µ_Al_(E), µ_Cu_(E) – linear attenuation coefficients for aluminium and copper, and x_Al_(V), x_Cu_(V) – thickness of the filters as per Table S1.


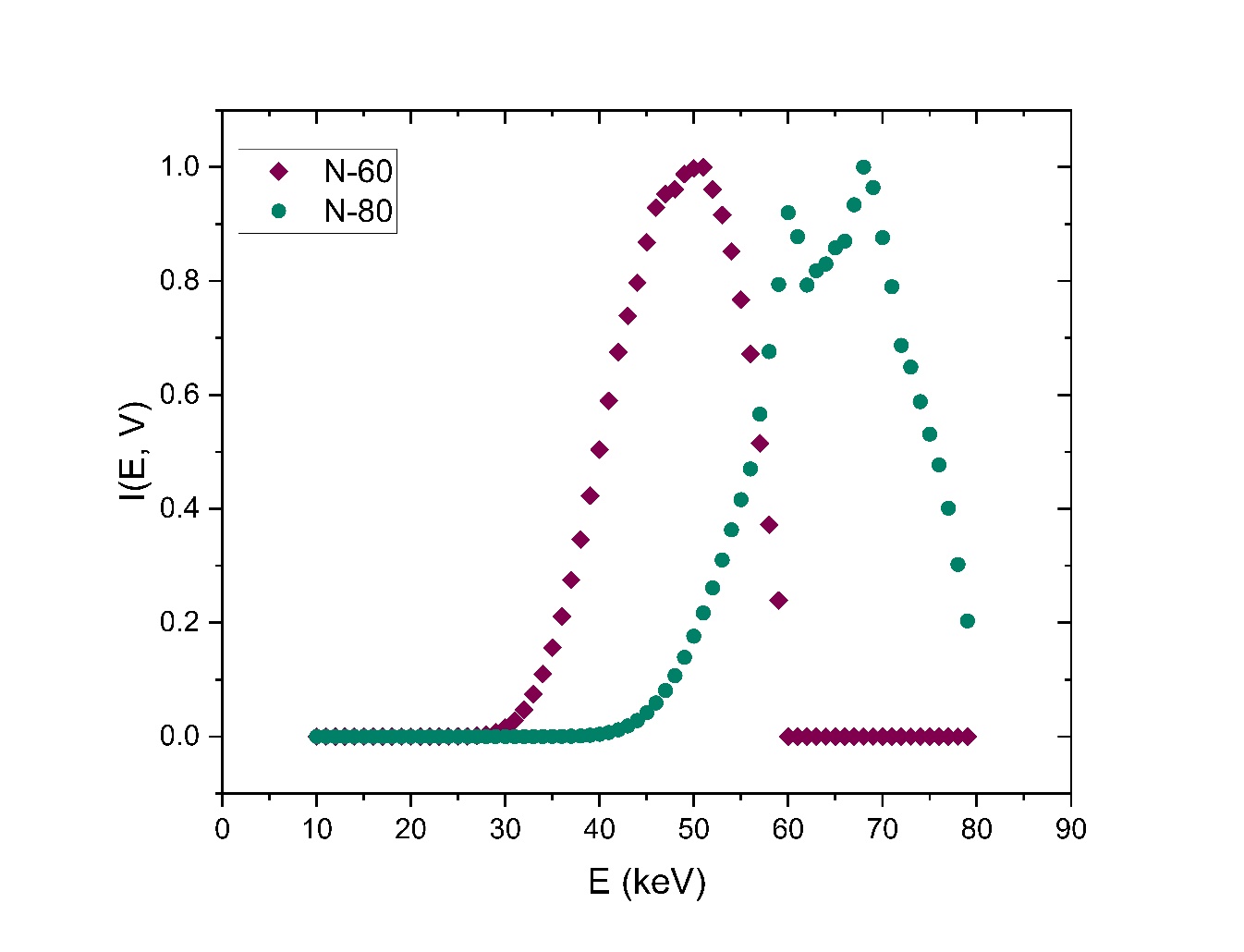


Figure S1. Calculated photon energy spectra (normalized) for two cases: N-60 and N-80 (see: Table S1).

The effective mass (or linear) attenuation coefficient for each of the standard radiation qualities was estimated as the mean of mass attenuation coefficients, weighted by radiation intensity at the corresponding energy level. Similarly, to before, the mass attenuation coefficient values for each point µ(E_i_) were sourced from the XCOM model:

| $\frac{\mu_{eff}}{\rho}\left( V \right)=\frac{\sum_{i}^{N} \frac{\mu}{\rho}\left( E_{i} \right)\cdot I\left( E_{i},V \right)}{\sum_{i}^{N} I\left( E_{i},V \right)}$ | (S2) |
| --- | --- |

where $\frac{\mu_{eff}}{\rho}\left( V \right)$ – estimated effective mass attenuation coefficient of a material for a particular radiation quality, $\frac{\mu}{\rho}\left( E_{i} \right)$ – mass attenuation coefficient of the material, I(E_I_,V) – radiation intensity given by Equation S1.

All calculations were performed using 70 energy values evenly distributed from 10 keV to 80 keV, see Supplementary Figure S1. All results are presented in Supplementary Table S2.

*Table S2. Results of the energy value assessment for two cases: N-60 and N-80, which were applied in Figure 1 (see main text). Results were presented both for mass and linear attenuation coefficient.*

| Material | Density  g/cm^3^ | N-60 | | N-80 | |
| --- | --- | --- | --- | --- | --- |
|  |  | $\frac{\mu_{eff}}{\rho}$  cm^2^/g | $\mu_{eff}$  cm^-1^ | $\frac{\mu_{eff}}{\rho}$  cm^2^/g | $\mu_{eff}$  cm^-1^ |
| ABS/GNPs composite | 1.064 | 0.189 | 0.201 | 0.174 | 0.186 |
| Aluminium | 2.699 | 0.373 | 1.007 | 0.230 | 0.621 |
| Lead | 11.35 | 9.087 | 103.133 | 3.978 | 45.146 |
| Pure Carbon | 1.700 | 0.177 | 0.301 | 0.163 | 0.277 |

**Review and comparison of different nanocomposite materials tested as radiation shields.**

As an additional verification of the effectiveness of gamma radiation shielding, the discussed material was compared with other conventional ones, i.e. aluminum, iron, copper and lead (see: Supplementary Figure S2). Measurements were carried out by the Department of Quality Control and Radiation Protection (UM) for three photon energies mentioned earlier: 122, 356, and 662 keV.


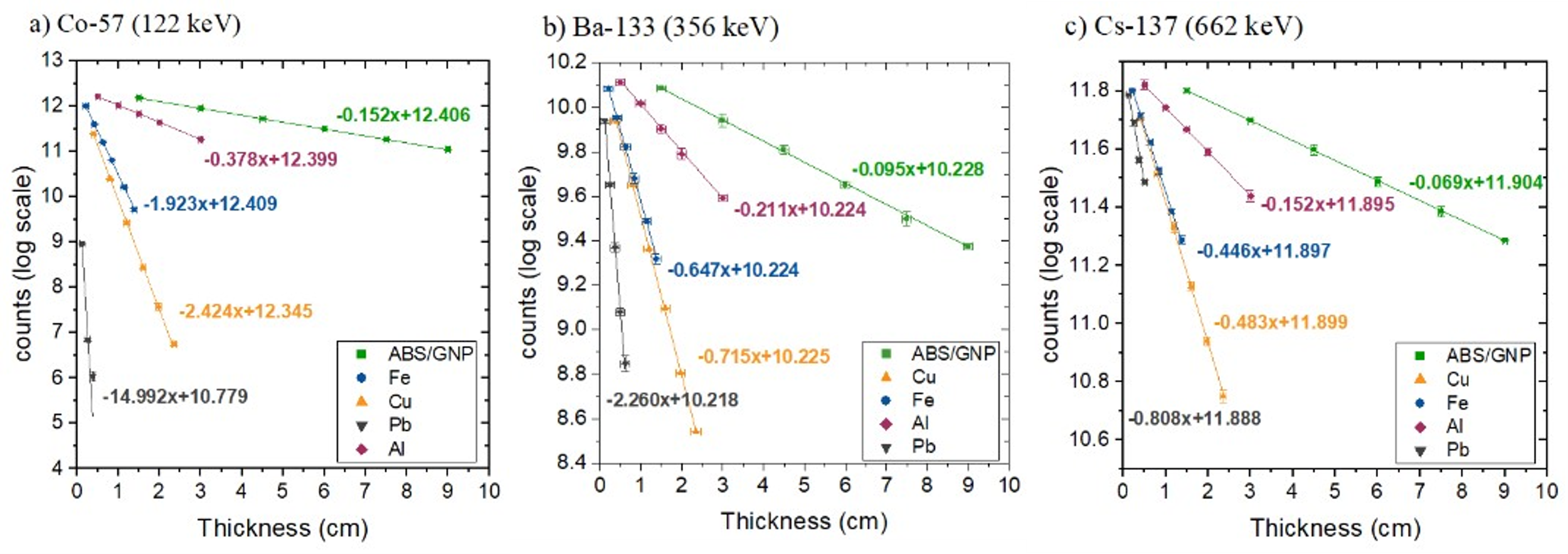


Figure S2. Graphene based nanocomposite shielding properties comparison to the materials i.e. aluminum, copper, iron, lead in a function of its thickness at photon energy of 122 keV (a), 356 keV (b) and 662 keV (c).

The Supplementary Table S3 summarizes the detailed information used for a Figure 3 (main text) comparing nanocomposites developed to date and materials commonly used for gamma and x-ray shielding.

*Table S3. Review and comparison of different nanocomposite materials tested as radiation shields.*

| No | Material | Density (g cm^-3^) |  | Linear attenuation coefficient (cm^-1^) | | | | Ref. |
| --- | --- | --- | --- | --- | --- | --- | --- | --- |
|  |  |  | 0.0479MeV | 0.0652MeV | 0.122 MeV | 0.356 MeV | 0.662 MeV |  |
| 1 | HDPE/Mo (10wt%) | 1.825 | 1.751 | 0.951 | - | - | - | ^34^ |
| 2 | HDPE/MoC (10wt%) | 1.749 | 1.522 | 0.911 | - | - | - |  |
| 3 | HDPE/W (10wt%) | 2.769 | 2.410 | 1.264 | - | - | - |  |
| 4 | HDPE/WC (10wt%) | 2.739 | 2.758 | 1.421 | - | - | - |  |
| 5 | Tungsten Carbide | 15.63 | - | - | - | 4.54 | 2.98 | ^9^ |
| 6 | Lead | 11.34 | - | - | - | 3.27 | 1.65 |  |
| 7 | Epoxy/WO_3_ (5wt%) | 1.66 | - | - | - |  | 0.121 | ^35^ |
| 8 | Epoxy/WO_3_ (10wt%) | 1.73 | - | - | - | - | 0.126 |  |
| 9 | Epoxy/WO_3_ (15wt%) | 1.81 | - | - | - | - | 0.130 |  |
| 10 | Epoxy/WO_3_ (20wt%) | 1.89 | - | - | - | - | 0.134 |  |
| 11 | Gelatin/MWCNT (10wt%) |  | - | - | - | - | 0.091 | ^36^ |
| 12 | Gelatin/Activated carbon (10wt%) |  | - | - |  | - | 0.087 |  |
| 13 | Bismuth oxychloride-filled polyester concretes (5wt%) | 1.322 | - | - | 0.370 | 0.141 | 0.106 | ^37^ |
| 14 | Bismuth oxychloride-filled polyester concretes (10wt%) | 1.383 | - | - | 0.510 | 0.163 | 0.110 |  |
| 15 | Bismuth oxychloride-filled polyester concretes (15wt%) | 1.408 | - | - | 0.663 | 0.166 | 0.120 |  |
| 16 | Bismuth oxychloride-filled polyester concretes (20wt%) | 1.429 | - | - | 0.850 | 0.179 | 0.121 |  |
| 17 | Polyacrylamide/ZnO (5wt%) | 1.335 | - | - | 0.214 | 0.142 | 0.110 | ^38^ |
| 18 | Polyacrylamide/ZnO (10wt%) | 1.559 | - | - | 0.261 | 0.166 | 0.127 |  |
| 19 | Polyacrylamide/ZnO (15wt%) | 1.784 | - | - | 0.312 | 0.189 | 0.145 |  |
| 20 | Polyacrylamide/ZnO (20wt%) | 2.008 | - | - | 0.365 | 0.213 | 0.162 |  |
| 21 | HDPE/ZnO (10wt%) | 1.042 | - | - | - | 0.113 | 0.089 | ^39^ |
| 22 | HDPE/ZnO (20wt%) | 1.104 | - | - | - | 0.124 | 0.096 |  |
| 23 | HDPE/ZnO (30wt%) | 1.232 | - | - | - | 0.132 | 0.102 |  |
| 24 | HDPE/ZnO (40wt%) | 1.282 | - | - | - | 0.142 | 0.106 |  |
| 25 | HDPE/PbO (10wt%) | 0.983 | - | - | - | 0.127 | 0.088 | ^40^ |
| 26 | HDPE/PbO (50wt%) | 1.507 | - | - | - | 0.301 | 0.156 |  |

**References:**

9. Jamal AbuAlRoos, N., Azman, M. N., Baharul Amin, N. A. & Zainon, R. Tungsten-based material as promising new lead-free gamma radiation shielding material in nuclear medicine. *Physica Medica* **78**, 48–57 (2020).

18. Alhindawy, I. G., Sayyed, M. I., Almuqrin, A. H. & Mahmoud, K. A. Optimizing gamma radiation shielding with cobalt-titania hybrid nanomaterials. *Sci Rep* **13**, 8936 (2023).

21. Berger, M. J. , *et al.* XCOM: Photon Cross Section Database (Version 1.5). *National Institute of Standards and Technology, Gaithersburg, MD* http://physics.nist.gov/xcom.

33. ISO 4037-1:2019 Radiological protection X and gamma reference radiation for calibrating dosemeters and doserate meters and for determining their response as a function of photon energy. Preprint at (2019).

34. Almurayshid, M., Alsagabi, S., Alssalim, Y., Alotaibi, Z. & Almsalam, R. Feasibility of polymer-based composite materials as radiation shield. *Radiation Physics and Chemistry* **183**, 109425 (2021).

35. Zali, V. S., Jahanbakhsh, O. & Ahadzadeh, I. Preparation and evaluation of gamma shielding properties of silicon-based composites doped with WO3 micro- and nanoparticles. *Radiation Physics and Chemistry* **197**, 110150 (2022).

36. Altarawneh, M., Aladailaha, M. & Al-Madanat, O. The Effect of Multi-Wall Carbon Nanotubes Addition on the Shielding Properties Against Gamma Radiation. *East European Journal of Physics* 524–530 (2023) doi:10.26565/2312-4334-2023-3-60.

37. Sharma, A. *et al.* Photon-shielding performance of bismuth oxychloride-filled polyester concretes. *Mater Chem Phys* **241**, 122330 (2020).

38. Nasehi Farnaz, I. M. Evaluation of X and Gamma-rays Attenuation Parameters for Polyacrylamide and ZnO Composites as Light Shielding Materials Using MCNP and X-COM Simulation. *J Nucl Med Radiat Ther* (2019).

39. Alsayed, Z., Badawi, Mohamed. S., Awad, R., El-Khatib, Ahmed. M. & Thabet, Abouzeid. A. Investigation of γ-ray attenuation coefficients, effective atomic number and electron density for ZnO/HDPE composite. *Phys Scr* **95**, 085301 (2020).

40. Mahmoud, M. E. *et al.* Fabrication, characterization and gamma rays shielding properties of nano and micro lead oxide-dispersed-high density polyethylene composites. *Radiation Physics and Chemistry* **145**, 160–173 (2018).

41. Boone, J. M. & Seibert, J. A. An accurate method for computer‐generating tungsten anode x‐ray spectra from 30 to 140 kV. *Med Phys* **24**, 1661–1670 (1997).
